# Virtual Reality to Support Inpatient Addiction Treatment: Patients Are Ready, What About Therapists? A Feasibility Study

**DOI:** 10.1007/s11469-022-00843-9

**Published:** 2022-07-28

**Authors:** Clemence Arissen, Laura van der Helm, Boukje Dijkstra, Wiebren Markus

**Affiliations:** 1 IrisZorg: Institution for Addiction Care, Sheltered Housing and Social Support Services, P.O. box 351, 6800 AJ Arnhem, The Netherlands; 2 NISPA: Nijmegen Institute for Scientist-Practitioners in Addiction, Radboud University, P.O. box 9104, 6500 HE Nijmegen, The Netherlands

**Keywords:** Feasibility, Virtual reality, Addiction care

## Abstract

This study aimed to identify facilitators and barriers for implementation of virtual reality therapy (VRT), used to train communication and problem-solving skills aiding relapse prevention, when integrated with addiction treatment (Treatment as Usual; TAU). Mixed methods were used in an observational, partly prospective, design. A total of 21 therapists and 113 patients from three inpatient addiction clinics were assessed. Therapists filled in questionnaires to gauge expectancies and experiences regarding facilitators and barriers at baseline, after a try-out period, halfway, and at the end of the pilot lasting 6–12 months. They also participated in focus-group interviews. Patients filled in similar questionnaires before an initial, and after they finished a third, VRT session. In addition, nine patients were interviewed. All VRT sessions were logged, with patients answering additional questions. Acceptability of VRT was high in both groups. It was feasible to integrate VRT with TAU and integration showed potential effectiveness. Barriers included incidental motion sickness, technical difficulties, costs, and device setup time. Both therapists and patients advocated VRT use to augment addiction treatment. Findings suggest a clinical effectiveness study is warranted.

In recent years, virtual reality therapy (VRT) has shown promise in a variety of psychological problems and psychiatric disorders. VRT has been applied in addiction care for the treatment of substance use and gambling (Langener et al., 2021; Segawa et al., 2020). Virtual reality is currently used both diagnostically, to assess cue reactivity (craving, physiological response or attentional bias), and therapeutically (VRT), in participants with tobacco, alcohol, and methamphetamine use disorder, as well as pathological gambling. In VRT, patients are exposed to stimuli similar to their experiences in the real world using a computer-generated virtual reality. VRT has been applied across paradigms including exposure therapy (e.g., Pericot-Valverde et al., 2019), CBT and social skills training (e.g., Ferreri et al., 2018), counterconditioning (using exposure to aversive stimuli or situations; e.g., Choi & Lee, 2015), and “embodied learning” (Langener et al., 2021), one example being crushing virtual cigarettes (Girard et al., 2009).

Characteristics of VRT make it an interesting approach when compared to regular treatment. Due to its immersive nature, VRT provides a form of experiential learning (Riva, 2022), whereas regular behavioral treatment relies more on verbal-cognitive abilities. This is a plus for patients with intellectual disabilities. VRT also allows users to practice gradually, replacing unwanted behaviors with desired ones, something harder to achieve with practice homework between sessions without the presence of a therapist (Nabors et al., 2020). Furthermore, VRT makes it possible to strengthen the self-efficacy by practicing a difficult situation (e.g., refusing drugs). Self-efficacy is the degree to which patients felt they could handle a (difficult) situation and has been identified to be an important determinant of health behavior change (Kadden & Litt, 2011). According to Bandura (1986), people who have both the necessary skills and strong coping efficacy, are likely to mobilize the effort needed to successfully resist situations of high-risk for drinking or drug use. VRT may also be useful for individuals who are reluctant to participate in in vivo or imaginary exposure (Bush, 2008). The ability to match virtual reality environments and patients’ preferences and needs positions VRT at the forefront of developments in personalized medicine (Segawa et al., 2020).

However, more research is needed before VRT can be embraced in clinical practice by therapists and patients. Acceptance of VRT among patients with non-addiction mental disorders seems favorable (Navarro-Haro et al., 2017). Segal et al. (2011) used an online questionnaire to study therapists’ (*N* = 271) perceptions of the advantages and disadvantages of VRT in psychotherapy, as compared to treatment as usual (TAU). Results indicated that therapists perceived the potential benefits to be greater than the potential costs. The highest rated benefit was the potential of VRT to expose clients to stimuli that are impractical or difficult to attain in reality. Therapists’ perceptions of the benefits of using VRT are related to their self-reported knowledge of virtual reality, interest in using virtual reality, and theoretical orientation. Cognitive-behavioral therapists gave more positive ratings than psychodynamic therapists. This is in accordance with virtual reality being used more frequently in cognitive behavioral therapy as a form of exposure therapy.

The acceptance of VRT as an adjunct to regular addiction treatment has not been studied. In addition, systematic assessment of VRT user experience is scarce. An innovation does not necessarily have to be a success in clinical practice. There can be many reasons why an innovation is not always used as intended. For instance, VRT uses in clinical practice may be hampered by factors such as accessibility (e.g., the costs of virtual reality hard- and software: Segal et al., 2011) and the idea that VRT use challenges professionals’ acquired knowledge and skills. Physiological barriers such as motion sickness and dry eyes, as well as psychological barriers, such as preoccupation and addiction to the VRT environment, have been described (Park et al., 2019). As a result, VRT may be underused by organizations, therapists, and patients. Therefore, a feasibility study was designed to identify relevant facilitators and barriers to the implementation of VRT, integrated with regular inpatient addiction treatment (TAU), from the perspective of both therapists and patients, using the Feasibility Framework described by Bowen et al. (2009).

## Methods

**Fig. 1:**
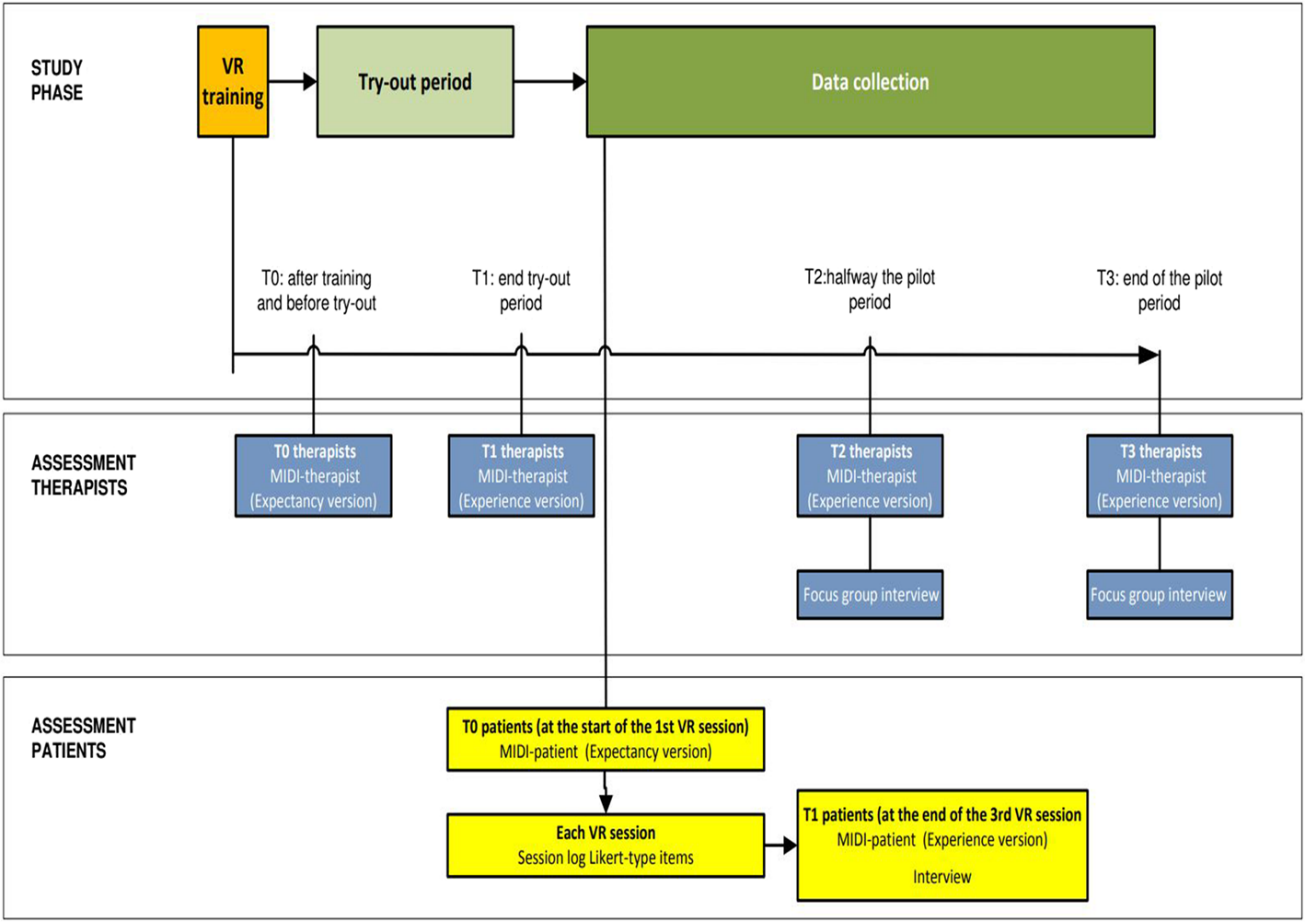
Overview of design, participant flow, and assessments

This feasibility study was observational in nature and had a prospective design (see Fig. 1). Using mixed methods, both participating therapists and patients were assessed multiple times. The study was approved by the Ethics Committee Faculty of Social Sciences of the Radboud University Nijmegen, the Netherlands (ECSW-2020-050R1).

### Participants

Data collection took place in three inpatient addiction facilities of IrisZorg (locations “Zevenaar,” “Wolfheze,” and “Tiel”), a center for addiction care and sheltered housing, located in the Netherlands. A total of 21 therapists were recruited to provide VRT. Eligibility criteria for the therapists were (1) employed in one of the participating clinics; (2) having concluded, or in the process of completing, training in TAU (Community Reinforcement Approach, CRA; Hunt & Azrin, 1973, an evidence-based addiction treatment); (3) completed VRT training provided by the VRT software developer (clevr.net); and (4) willingness to participate in monthly meetings with their participating colleagues and the researchers involved.

A total of 113 patients receiving inpatient addiction treatment at one of the participating sites were recruited between October 2020 and October 2021. Eligibility criteria for the patients were (1) age 18 years or older and (2) a diagnosis of substance use disorder according to *DSM-5* criteria (American Psychiatric Association, 2013). Exclusion criteria included (1) a current, known diagnosis of epilepsy; (2) known sensitivity to flashing lights or motion, (3) vision that needed correction with glasses whereby the glasses, due to their size, prohibited the use of the virtual reality headset; and (4) recent injury to the eyes, face, neck, or arms that prevented comfortable use of virtual reality.

### Intervention

VRT was provided using software developed by CleVR (clevr.net). Its dynamic, interactive features allow the therapist to interact directly with the patient in a customizable virtual environment of choice varying in context (e.g., shopping street, at home, café, office), position (e.g., sitting, walking, or standing), and background sounds and messages (e.g., music, advertising, traffic), and using one or more virtual agents (females, males, and children) that can be customized in appearance, height, and voice. The therapist controls the agents (one at a time) via an interface on a tablet, and can use this to influence the emotional (facial) expression, hand gestures, bodily behavior, and verbal expression of the active agent. The therapists’ voice can be distorted to provide a unique pitch to each of the agents’ voices. The therapist is also able to see (via laptop screen) what the patient sees when wearing the VRT-headset. The therapist communicates with the patient through a microphone while the patient wears a headphone. The patient stands or is seated when wearing the VRT-headset, depending on the VRT environment.

In this study, the role-play function of the Social Worlds 4.0 software was used to practice communication- and problem-solving skills (e.g., substance refusal), analogue to the role-play training provided as part of TAU. So VRT was used as an extension of regular, group-wise CRA training. When VRT was provided to pairs of patients, the patient who did not wear the VRT-headset was instructed to watch the interactions during VRT via laptop screen and to provide feedback after each practice round. During each session, time was taken to make a shared decision on the per-session training objective and to tailor the virtual environment and agent(s) to the needs of that particular patient (such that these reflected their real-life experience or expectations to a certain degree). Next, patients practiced their skills in a scenario they anticipated fearing in the near future (e.g., saying no to old friends who try to persuade the patient to have a drink with them). After 1–3 min of role play, the patient was invited to reflect on his performance, and if needed received feedback and tips for a stronger performance, after which they re-entered the scenario. This cycle was repeated several rounds, until patients demonstrated the desired behavior to their own and the therapists’ satisfaction.

### Instruments

#### Measuring Instrument Determinants of Innovations (MIDI)

Questionnaires for therapists and patients to determine expectancies and experiences regarding facilitators and barriers were based on the Measuring Instrument Determinants of Innovations instrument (MIDI; Fleuren et al., 2014). The MIDI provides a framework to evaluate the implementation of health care innovations. It distinguishes four groups (innovation-, professional-, organization-, and socio-political-related) of determinants that may facilitate or impede implementation of innovations. Most of the 29 determinants are measured by one or multiple Likert-type scale items with scores of 0 (“Totally disagree”) to 5 (“Totally agree”). Depending on the implementation or innovation, the user is encouraged to adapt the wording of items and selection of relevant determinants.

The selection of MIDI determinants for this study was guided by Bowen et al. (2009), who identified eight general focus areas addressed by feasibility studies: “Acceptability,” “Demand,” “Implementation,” “Practicality,” “Adaptation,” “Integration,” “Expansion,” and “Limited-efficacy.” Five focus areas could be matched with 20 selected MIDI determinants (see Table 1). In this study, MIDI versions “Expectations” (immediately after the dynamic interactive virtual reality training) and “Experiences” (after a 2- to 4-week try out period, halfway, and at the end of the data collection phase) were prepared for the participating therapists (6 and 24 items, respectively). In our sample, the internal consistency of the MIDI questionnaire for patients was good–excellent (total scale: *α* = 0.860, patients expectations: *α* = 0.913, patients experiences: *α* = 0.880). Furthermore, the internal consistency of the MIDI questionnaire for therapists was good–excellent (total scale: *α* = 0.847, therapists expectations: *α* = 0.772, therapists experiences: *α* = 0.864). See the Appendix for more details.

**Table 1:** Overview of general areas of focus in feasibility studies and their operationalization

General area of focus in feasibility studies (Bowen et al., 2009)	Relevant MIDI determinants (Fleuren et al., 2014)	MIDI therapist expectations (directly following training)	MIDI therapist experiences (3: after try-out, halfway, and end)	Other sources of therapist information	MIDI patient expectations (start 1st session)	MIDI patient experiences (end 3rd session)	Other sources of patient information
1. Acceptability	Correctness (MIDI determinant 2; 1 item)		X	Focus group interviews		X	Interviews
	Compatibility (MIDI determinant 5; 1 item)		X			X	
	Relevance for patient (MIDI determinant 7; 1 item)	X			X		
	Personal benefits/drawbacks (MIDI determinant 8; 12 items)		X			X	
	Professional obligation (MIDI determinant 10; 1 item)		X			X	
	Patient satisfaction (MIDI determinant 11; 1 item)	X				X	
	Patient cooperation (MIDI determinant 12; 1 item)	X			X		
2. Demand				Interviews			Focus group interviews
3. Implementation	Complexity (MIDI determinant 4, 1 item)		X	Focus group interviews		X	Focus group interviews
	Social support (MIDI determinant 13; 4 items)		X				
4. Practicality	Completeness (MIDI determinant 3; 2 items)		X				Focus group interviews
	Knowledge (MIDI determinant 17; 2 items)		X				
	Staff capacity (MIDI determinant 21; 1 item)		X				
	Time available (MIDI determinant 23; 1 item)		X				
	Material resources and facilities (MIDI determinant 24; 1 item)		X				
	Information accessible about use of the innovation (MIDI determinant 27; 1 item)		X				
	Performance feedback (MIDI determinant 28; 1 item)		X				
5. Adaptation	Procedural clarity (MIDI determinant 1; 2 items)		X	Focus group interviews			Interviews
6. Integration				Focus group interviews			
7. Expansion				Focus group interviews			Interviews
8. Limited-efficacy	Observability (MIDI determinant 6; 1 item)		X	Focus group interviews		X	Interviews VRT-session log
	Outcome expectations (MIDI determinant 9; 6/11 items)	X			X		
	Self-efficacy (MIDI determinant 16; 3 items)	X					

#### Focus Groups and Individual Interviews

The topic list for focus groups with therapists was based on all of Bowen et al.’s (2009) focus areas. In addition, patients were interviewed by telephone. The topic lists for these interviews were based on Bowen et al.’s (2009) focus areas: “Acceptability,” “Demand,” “Adaptation,” “Expansion,” and “Limited-efficacy.”

#### VRT Session Log/Questionnaire

During each VRT-session, the following components were logged: whether VRT was provided to an individual or in a group, the skill that was practiced, to what extent patients experienced reduced symptoms since the previous session due to the use of VRT, and whether they had practiced the skills trained in VRT since the previous session, as well as their self-efficacy: the degree to which patients felt they could handle the practice situation prior to and after the practice cycle in the virtual environment, all on Likert-type scales from 0 (“Not at all”) to 10 (“Maximal”). At the end of each session, patients rated items regarding Bowen et al.’s (2009) focus areas: “Limited-efficacy” and “Acceptability” of the VRT.

### Procedure

#### Therapists

Eligible therapists who gave informed consent received a day-long, group VRT training by the VRT software developer (CleVR), aimed at being able to operate the VRT hard- and software. In order to provide therapists with tools to shape the VRT sessions, we developed scripts for the CRA modules substance refusal, communication skills, problem solving, relapse management, and weekend leave preparation. This concerns “translation” of existing intervention descriptions into the application of that same intervention with the support of VRT.

The therapists of each participating site received monthly group intervision with the first, second, and/or last author. The goal was to provide guidance to help overcome obstacles, to facilitate creative applications of VRT in clinical practice beyond those stated a priori, and, if necessary, to repeat instructions on data collection. After a try-out period of 2 to 4 weeks in which therapists practiced VRT on each other, they started to provide VRT to inpatients who had been receiving TAU. Therapists were encouraged to experiment with the timing, intensity, frequency, and duration of the VRT sessions, with integration with TAU, and the number of therapists and patients per VRT session that seemed optimal. It was thought that this allowed for optimal creativity as well as therapist (and possibly patient) engagement.

After the training, at the end of the try-out phase, halfway, and at the end of the data-collection period, therapists filled in the MIDI questionnaires aimed at assessing expectancies (baseline only) and experiences (all other time-points). At the latter two time-points, therapists also participated in a focus group interview (see Fig. 1).

#### Patients

In inpatient facility “Zevenaar,” eligible patients were recruited and instructed in the first week following admission, after 1 week of detoxification. After receiving at least 1 week of CRA group training, three 90-min VRT sessions were planned for the following weeks, spaced 1–4 days apart. During the VRT sessions one or two patients were trained simultaneously by one therapist, sometimes two.

In inpatient facility “Wolfheze,” eligible patients were screened after following the basic CRA modules during the first few weeks after admission and after consultation with the director of the treatment program. Then, in one or more individuals, 60-min VRT sessions were scheduled in the following weeks, spaced 1–7 days apart. In these sessions, individual patients were trained by one therapist. This was done because the patient population at this site exhibited more complex psychopathology (including psychotic disorders) and it was felt they required a more tailored approach.

In inpatient facility “Tiel,” eligible clients could participate in VRT sessions immediately after detoxification. Therapists initially started using VRT in a group of eight adolescents, accompanied by two therapists. It was observed that patients lost their concentration and took VRT less seriously, however. Subsequently, individual patients were trained by two therapists. In one or more individuals, 60-min VRT sessions were scheduled in the following weeks, spaced 1–7 days apart.

Patients from all sites filled in the MIDI questionnaires at the start of the first (assessing expectancies) and at the end of the third VRT session (if provided, assessing experiences). During each session, information about the session, the experiences with, and the results by VRT was measured (see VRT session log/questionnaire).

A total of nine patients who experienced at least three VRT sessions were approached for an additional semi-structured interview after their last VRT session. Recruitment for individual interviews ended after data saturation was reached.

### Statistical Analyses

Quantitative data were analyzed in SPSS statistics, v27 (IBM, Armonk, NY, 2020). Tables with counts, percentages, means, standard deviations, and ranges were used to gain an overview of descriptive variables.

In line with Verberne et al. (2018), MIDI items where ≥ 20% of participants scored “Disagree” or “Strongly disagree” were flagged as barriers, while items where ≥ 80% of participants scored “Agree” or “Strongly agree” were flagged as facilitators for implementation. Items that score between 20 and 80% were flagged as neutral. MIDI item 4 and some questions under MIDI item 8 were recoded for this purpose. Subsequently, descriptive statistics were calculated with regard to MIDI items reflecting expectancies and experiences aimed at both therapists and patients.

Qualitative data (focus group and individual interviews) were transcribed. A pre-established code tree (a combination of main and sub-codes), based on the topic lists from Bowen et al. (2009), was used to analyze the interview data. By taking the topic lists as a starting point and thus also the code tree, the data could be analyzed efficiently to identify relevant facilitators and barriers to the implementation of VRT.

Paired *t*-test was used, with an alpha level of 0.05 (based on 95% confidence interval), to measure the difference to which the patients felt they could handle the practice situation prior to and after VRT practice (self-efficacy), based on mean scores from the Likert-type scale from each VRT-session.

## Results

### Sample Characteristics

Therapists (*N* = 21) ranged in age from 23 to 53 years (*M* = 33.32, *SD* = 9.01), a total of 76% female. Most therapists (53%) worked as a social therapist while 47% worked as a psychologist. A total of 37% received basic CRA training, 37% received additional coaching, and 26% finished additional CRA coaching. Some (16%) had 1 year working experience with CRA, while 42% had 2 years of CRA experience, and 42% had more than 3 years of CRA working experience.

Patients (*N* = 113) ranged in age from 21 to 72 years (*M* = 41.60, *SD* = 11.83). A total of 29% of patients were female. About 36% had obtained a high school diploma and 3% had a university degree. For 61%, the level of education was missing from the patient records. All patients were diagnosed with a substance use disorder, according to DSM-5 criteria. Known comorbid diagnoses were personality disorders (27%), anxiety or mood disorders (11%), neurodevelopmental disorders (39%), schizophrenia spectrum/other psychotic disorders (5%), trauma- and stressor-related disorders (18%), and neurocognitive disorders (5%). The number of previous inpatient treatments ranged from 0 to 13 (*M* = 2.11, *SD* = 2.84).

### Indicators of Feasibility: Facilitators and Barriers for the Implementation of VRT from the Therapists’ Perspective

#### MIDI Questionnaires: Therapists

##### Expectations

When therapists rated their expectations toward VRT, facilitators were identified for focus areas “Acceptability” (*M* = 4.03, *SD* = 0.39) and “Limited-efficacy” (*M* = 4.26, *SD* = 0.38). The specific MIDI determinants are listed in Table 2. These results implied that therapists had high expectations about the suitability of VRT, about patient satisfaction when using VRT, and about patient cooperation when therapists used VRT as treatment modality. Therapists also had high expectations of their patients when it comes to being better able to refuse drugs, having developed better communication skills, being better prepared for weekend leave because risk situations have been practiced, and being better able to handle risk situations because of these having been practiced. Therapists also expected to be well positioned to explain the added value of VRT and to motivate the patient to use VRT.

**Table 2:** Facilitators and barriers according to therapists

General areas of focus in feasibility studies (Bowen et al., 2009)	Relevant MIDI determinants and items (Fleuren et al., 2014)	MIDI questionnaire type	Percent^a,b^ agreement	Type of qualification
1. Acceptability	**MIDI determinant 2: Correctness**			
	Item 1: The use of VRT in addition to the regular CRA treatment is well substantiated	Experience	**88.4%**	**Facilitator**
	**MIDI determinant 5: Compatibility**			
	Item 1: VRT fits in well with the regular CRA treatment	Experience	61.6%	Neutral
	**MIDI determinant 7: Relevance patient**			
	Item 1: VRT is suitable for most of my patients	Expectation	**94.8%**	**Facilitator**
	**MIDI determinant 8: Personal benefits/drawbacks**			
	Item 1: It makes my treatments more fun	Experience	**92.7%**	**Facilitator**
	Item 2: It makes me more attentive to clients’ skills needed for homework, leave, etc		67.5%	Neutral
	Item 3: It gives me more opportunities to help patients		**91.9%**	**Facilitator**
	Item 4: It gives a better return than homework assignments alone		76.3%	Neutral
	Item 5: It fits better than regular role-playing games		**80.5%**	**Facilitator**
	Item 6: Patients become more skilled		**92.0%**	**Facilitator**
	Item 7: I experience an extra burden in time		51.2%	Neutral
	Item 8: I find the equipment and software complicated to work with		49.9%	Neutral
	Item 9: I find it difficult to take an active role in VRT		**82.9%**	**Facilitator**
	Item 10: It makes treatments unnecessarily complex		**94.7%**	**Facilitator**
	Item 11: Clients often do not want to work with it		0.0%	Neutral
	Item 12: The extra time and effort do not outweigh the possibilities and effect of VRT		60.2%	Neutral
	**MIDI determinant 10: Professional obligation**			
	Item 1: I think it is part of the function of (CRA) therapists to use VRT in the CRA treatment	Experience	43%	Neutral
	**MIDI determinant 11: Patient satisfaction**			
	Item 1: Patients will generally be satisfied if I use VRT	Expectation	**84.2%**	**Facilitator**
	**MIDI determinant 12: Patient cooperation**			
	Item 1: Patients will generally cooperate if I use virtual reality in treatment	Expectation	**84.5%**	**Facilitator**
3. Implementation	**MIDI determinant 4: Complexity**			
	Item 1: VRT is too complicated for me to use	Experience	34.1%	Neutral
	**MIDI determinant 13: Social support**			
	Item 1: I can count on the help of my organization, my location coordinator/ management if I need it when using VRT:	Experience	34.8%	Neutral
	Item 2: The service desk of CleVR		**98.3%**	**Facilitator**
	Item 3: The project members of the feasibility study		**87.7%**	**Facilitator**
	Item 4: My trained colleagues		**89.9%**	**Facilitator**
4. Practicality	**MIDI determinant 3: Completeness**			
	Item 1: CleVR’s training and service offers all the information and materials needed to work well with the virtual reality set	Experience	**93.1%**	**Facilitator**
	Item 2: The VRT manual provides all the information and materials needed to work properly with VRT in CRA treatment		**85.6%**	**Facilitator**
	**MIDI determinant 17: Knowledge**			
	Item 1: I have sufficient knowledge to be able to use VRT in treatment	Experience	**82.7%**	**Facilitator**
	Item 2: I believe you need to be CRA coded to properly apply VRT in CRA treatment		39.4%	Neutral
	**MIDI determinant 21: Staff capacity**			
	Item 1: There is sufficient staff in the clinic where I work to be able to use VRT as intended	Experience	63.5%	Neutral
	**MIDI determinant 23: Time available**			
	Item 1: IrisZorg makes enough time available for me to integrate VRT into my daily work as intended	Experience	69.3%	Neutral
	**MIDI determinant 24: Material resources and facilities**			
	Item 1: IrisZorg and CleVR will make sufficient materials and facilities available to me to be able to use VRT as intended	Experience	**95.8%**	**Facilitator**
	**MIDI determinant 27: Information accessible about use**			
	Item 1: I have easy access within IrisZorg (possibly CleVR) to information about the use of VRT	Experience	71.1%	Neutral
	**MIDI determinant 28: Performance feedback**			
	Item 1: Regular feedback will take place within IrisZorg about the progress of the feasibility study	Experience	69.7%	Neutral
5. Adaptation	**MIDI determinant 1: Procedural clarity**			
	Item 1: The VRT manual clearly states which activities I have to perform in which order when using the hardware	Experience	**85.7%**	**Facilitator**
	Item 2: The scripts clearly state which activities I need to perform in which order during the role plays		13.9%	Neutral
8. Limited-efficacy	**MIDI determinant 6: Observability**			
	Item 1: I find the effects of using VRT clearly noticeable	Experience	59.8%	Neutral
	**MIDI determinant 6: Outcome expectations**			
	Item 1: I believe it is important to use VRT to achieve the following goals with my client: Patients will feel more resilient in refusing drugs	Expectation	**94.8%**	**Facilitator**
	Item 2: Patients will have better communication skills		**100%**	**Facilitator**
	Item 3: Patients will be better prepared for leave because risk situations have been practiced		**100%**	**Facilitator**
	Item 4: I expect that with VRT, the following goals will actually be achieved with my patient: to feel more resilient in refusing drugs		**94.8%**	**Facilitator**
	Item 5: To have better communication skills		**89.9%**	**Facilitator**
	Item 6: Will be better prepared for leave because risk situations will have been practiced		**94.7%**	**Facilitator**
	**MIDI determinant 16: Self-efficacy**			
	Item 1: Explain to patients what (the added value of) VRT is and motivate them to use VRT in their treatment?	Expectation	**94.4%**	**Facilitator**
	Item 2: Work with the equipment and the controls (interface)?		73.7%	Neutral
	Item 3: Work with the scripts?		73.7%	Neutral

^a^MIDI cut-off scores therapists’ facilitators ≥ 80% agreement or barriers ≥ 20% disagreement

^b^Boldface is a facilitator

##### Experiences

When therapists rated their VRT experiences, facilitators were identified for areas of focus “Acceptability” (*M* = 3.43, *SD* = 0.18), “Implementation” (*M* = 3.78, *SD* = 0.319), “Practically” (*M* = 3.82, *SD* = 0.31), “Adaptation” (*M* = 3.82, *SD* = 0.32), and “Limited-efficacy” (*M* = 3.59, *SD* = 0.55). The specific MIDI determinants can be found in Table 2. Therapists found that VRT fitted in well with the regular CRA treatment. They experienced several personal benefits: it made the treatment more fun, it provided more possibilities to help patients, it fitted better than role play, and patients became more social skilled. Moreover, therapists did not find it difficult to take an active role, nor did VRT render treatment unnecessarily complex. Therapists experienced a lot of social support from the service desk, researchers, and trained colleagues. Also, the training and the manual were experienced as supportive, and therapists experienced that they had sufficient knowledge to apply VRT.

#### Focus Groups Interviews with Therapists About Their Experiences

Therapists reported integrating VRT with regular treatment as a fun challenge. It helped them to gain insight into the patient’s strengths and weaknesses, which also allowed the therapist to better adapt to them and slowly increase exposure intensity. “With VRT you just do it instead of talking about how you would do it,” one therapist remarked. Therapists saw, after 12 months of using VRT, the added value for their patients. Therapists saw that patients perceived VRT as realistic, with similar stresses as in real life: “You can tell by the patient’s body posture and that he is wiping his clammy hands on his pants.” VRT helped patients to obtain a realistic view of their skills and to gain insight into what still needs practice. It allowed to practice communication and problem-solving skills, needed to prevent relapse (e.g., substance refusal) in a realistic manner and continue to practice the skill until the patient had mastered it sufficiently. It also countered avoidance by patients. It aided in connecting with a part of the targeted patient group that had difficulty verbalizing their experiences. In addition, patients were not distracted by external stimuli when wearing the VRT glasses. Therapists found that working with VRT in a group of two patients had added value over individual VRT; the patient learned to provide feedback and often had an eye for other issues than the therapist did. At the end of the pilot, therapists concluded that VRT was more intense and thorough compared to role play. A patient could more easily separate the therapist from the role he or she played in VRT compared to an analogue role-play. The latter, on the other hand, was more interactive in a larger group, feedback could be given more ad hoc, and more fellow patients were engaged. Therapists therefore recommended that both forms continue to be used alongside each other. Barriers to VRT were the time investment, difficulty in scheduling therapists, and a single patient reported motion sickness (focus area “Acceptability”).

Therapists saw several applications of VRT besides training social skills, such as training emotion recognition in patients with intellectual disabilities, changing perspectives in personality problems, use as a diagnostic instrument (e.g., assessing what a patient needed to learn), and enhancing staff de-escalation skills. Knowing the VRT hard- and software well and regular use with patients is essential to continue using VRT as a part of overall treatment strategies. This is mentioned as a potential barrier if time is scarce (focus area “Demand”).

Therapists experienced no pressure from management, but also no specific support is mentioned. The administration of the questionnaires and formats used in this pilot was perceived as labor-intensive and a strain and as such constituted a barrier (focus area “Implementation”).

Proximity to a treatment room where the device is set up, a set schedule for VRT sessions, and sufficient time to prepare the exercises were all important conditions for optimal use of VRT. At one location, experiments were conducted with a group of up to eight adolescents with VRT. The considerable duration of the session and group dynamics led to taking VRT less seriously. Insufficiently trained VRT therapists, device failure, and the time required to set up and check the device (≥ 15–30 min) were perceived as barriers (focus area “Practically”).

Regarding “Adaptation,” therapists deployed VRT to the patient’s personally set goal, scripts proved unnecessary.

Completion by patients of the CRA communication skills module was a prerequisite for starting VRT. This way, therapists could refer to feedback rules and other communication skills during VRT. In general, VRT was considered broadly applicable within mental health and social care (focus area “Integration”).

Specific concerns mentioned by the therapists working in an inpatient setting included adapting VRT to the relative complex target population. That is, guard against overloading patients, aiming for small steps regarding social skills training in VRT. A relatively limited length of admission in one of the clinics calls for careful phasing of VRT deployment. No barriers were mentioned (focus area “Expansion”).

Finally, with regard to “Limited-efficacy,” through repetition of role play focusing on specific scenario’s in VRT, therapists noticed a growth in skills in their patients. Patients became more aware of their own influence on their environment. A tendency to externalize conflicts decreased through direct feedback. Patients seemed to get a more realistic view of their capabilities. Therapists felt that the use of VRT raised self-awareness and initiated behavioral change in patients. A thorough preparation of the target session (e.g., discussing the triggers and choice of words fitting the patient) was experienced as a barrier by therapists given the time involved. No differences between the locations were found.

### Indicators of Feasibility: Facilitators for and Barriers to the Implementation of VRT from the Patients’ Perspective

#### MIDI Questionnaire: Patients

##### Expectations

When patients rated their expectations, they scored neutral on the area of focus “Acceptability” (*M* = 3.80, *SD* = 0.04). The specific MIDI determinants could be found in Table 3. Overall, patients had no clear expectations regarding VRT use. They named one item as facilitator (80% score): “the expectation that patients will be better prepared for leave because risk situations will have been practiced.”

**Table 3:** Facilitators and barriers according to patients

General areas of focus in feasibility studies (Bowen et al., 2009)	Relevant MIDI determinants and items (Fleuren et al., 2014)	MIDI questionnaire type	Percent^a,b^ agreement	Type of qualification
1. Acceptability	**MIDI item 2: Correctness**			
	Item 1: The use of VRT in addition to the regular CRA treatment is well substantiated	Experience	**88.2%**	**Facilitator**
	**MIDI item 5: Compatibility**			
	Item 1: VRT fits in well with the regular CRA treatment	Experience	**88.0%**	**Facilitator**
	**MIDI item 7: Relevance patient**			
	Item 1: VRT is suitable for me and most of my patients	Expectation	55.8%	Neutral
	**MIDI item 8: Personal benefits/drawbacks**			
	Item 1: It makes my treatment more fun	Experience	**89.7%**	**Facilitator**
	Item 2: It makes me pay more attention to the skills I need for homework, leave, etc		**82.8%**	**Facilitator**
	Item 3: It gives more opportunities to be helped		**98.6%**	**Facilitator**
	Item 4: It gives a better return than just homework assignments		**86.2%**	**Facilitator**
	Item 5: It fits better than regular role playing		**96.4%**	**Facilitator**
	Item 6: I’m getting more skilled		**86.2%**	**Facilitator**
	Item 7: I experience side effects such as dry eyes or motion sickness (nausea, headache, sweating, fatigue, disorientation, etc.)		20.7%	Barrier
	Item 8: I find the equipment and software complicated to work with		78.5%	Neutral
	Item 9: I find it difficult to actively play a role in VRT		**82.7%**	**Facilitator**
	Item 10: It makes treatments unnecessarily complex		**82.8%**	**Facilitator**
	Item 11: Co-patients often don’t want to work with it		42.8%	Neutral
	Item 12: The extra time and effort does not outweigh the possibilities and effect of VRT		**85.2%**	**Facilitator**
	**MIDI item 10: Professional obligation**			
	Item 1: I think it is part of the function of (CRA) therapists to use VRT in the CRA treatment	Experience	45.8%	Neutral
	**MIDI item 11: Satisfaction patient**			
	Item 1: I am generally satisfied when using VRT	Experience	**93.1%**	**Facilitator**
	**MIDI item 12: Cooperation patient**			
	Item 1: Patients will cooperate	Expectation	77.6%	Neutral
3. Implementation	**MIDI item 4: Complexity**			
	Item 1: VRT is too complicated for me to use	Experience	**93.1%**	**Facilitator**
8. Limited-efficacy	**MIDI item 6: Observability**			
	Item 1: I find the effects of using VRT clearly noticeable	Experience	**82.1%**	**Facilitator**
	**MIDI item 6: Outcome expectations**			
	Item 1: That I feel more resilient in refusing drugs	Expectation	71.2%	Neutral
	Item 2: That I have better communication skills		75.0%	Neutral
	Item 3: That I am better prepared for leave because risk situations have been practiced		70.0%	Neutral
	Item 4: That patients feel more resilient in refusing drugs		72.3%	Neutral
	Item 5: That patients have better communication skills		75.1%	Neutral
	Item 6: That patients are better prepared for leave because risk situations have been practiced		**80.0%**	**Facilitator**
	Item 7: This type of treatment seems logical to me		58.9%	Neutral
	Item 8: I am sure that this treatment successfully contributes to the cessation of substance use		43.1%	Neutral
	Item 9: I would recommend this treatment to a friend who has the same addiction issues as me		54.2%	Neutral
	Item 10: I am willing to follow a CRA treatment with VRT because of my addiction		79.5%	Neutral
	Item 11: I think the use of VRT can also be successful in treating other types of problems, e.g., anxiety		75.6%	Neutral

^a^MIDI cut-off scores therapists’ facilitators ≥ 80% agreement or barriers ≥ 20% disagreement

^b^Boldface is a facilitator

##### Experiences

When patients rated their experiences, facilitators were identified for areas of focus “Acceptability” (*M* = 4.14, *SD* = 0.09) “Implementation” (*M* = 4.55, *SD* = 0.74), and “Limited-efficacy” (*M* = 3.83, *SD* = 0.03). The specific MIDI determinants could be found in Table 3. In summary, patients found that VRT fitted well in the regular CRA treatment. They reported a number of personal benefits: virtual reality made treatment more fun, it offered more possibilities to be helped, it fitted better than role play, and patients became more skilled. Above all, patients indicated that they were satisfied with the use of VRT, they found it not too complicated to use, and effects were noticeable.

#### Interview by Telephone

Patients reported that VRT helps them better cope with difficult situations. They found that VRT approximated reality through the ability to customize the virtual agents by gender, height, skin color, and appearance. It allowed repeated practice until the skill is mastered. Patients felt that avoidance is impossible. They also indicated that they experience barriers because they were expected to wear a paper mask due to COVID-19 measures which also required additional cleaning of the VRT set (focus area “Acceptability”).

Patients wanted to do everything they could to address their addiction and comorbid problems; therefore, they were motivated to use VRT as part of a broader treatment. The enthusiasm of fellow patients was also experienced as motivating. Patients, like therapists, saw applications of VRT in several areas: training resilience, social skills, conflict management, and practicing difficult conversations with agencies or job interviews, as well as exposure (focus area “Demand”).

Thorough goal preparation, using specific (triggering) words, and setting up the right practice situation in VRT seem essential for patients to make progress. The vast majority of patients used VRT to train their communication skills. Patients reported that they perceived the lack of certain virtual reality environments (garden, patio, standing table) as barriers (focus area “Adaptation”).

Patients indicated that they also saw possibilities for using the VRT set in the outpatient settings or within a social care domain. They suggested the use of VRT in aftercare, to practice communication skills and substance refusal for a longer period following discharge from a clinic (area of focus “Expansion”).

Finally, regarding the “Limited-efficacy” area of focus, all patients indicated that VRT helped them to make noticeable steps toward achieving their goals. The realistic character as well as the possibility of practicing goal behaviors until fully mastered, without the task becoming boring, was felt to help generalize the skills trained.

#### VRT Session Log

Patients, on average, indicated that VRT helped them achieve the goals they had practiced in VRT (*M* = 7.06, *SD* = 1.74) and that they planned to apply the practiced skill in practice during the coming period (*M* = 7.66, *SD* = 1.96). Regarding the latter, this also seems to have been followed through as indicated at the next session (*M* = 6.47, *SD* = 1.96). Patients were clearly satisfied with the use of VRT directly following the sessions (*M* = 7.92, *SD* = 1.34). The complaints or problems practiced seem to be favorably affected by VRT to some extent (*M* = 4.96, *SD* = 3.25).

Patients reported self-efficacy (*n* = 73) increased significantly from before (*M* = 6.70, *SD* = 1.78) to after VRT practice (*M* = 7.66, *SD* = 1.24), *t*(72) =  − 4.719, *p* < 0.001 on a Likert-type scale of 0 (“Not at all”) to 10 (“Maximal”).

## Discussion

The aim of this study was to identify relevant facilitators and barriers to the implementation of VRT when integrated with inpatient addiction treatment, following the Feasibility Framework by Bowen et al. (2009). Findings from this study showed that VRT integrated with TAU had a high acceptance rate in both therapists and patients. This was reflected in the finding that many facilitators were identified together with only a single barrier. Therapists had high expectations regarding VRT use. Patients did not have clear expectations at the outset, apart from anticipating a skill boost. The difference can be due in part to therapists having been trained for the use of VRT, while patients were more or less confronted with the option to use VRT, making it difficult for them to express their expectations. This pleads for a general introduction to VRT before starting VRT.

Acceptance rates remained high among both therapists and patients throughout the study. Participants viewed the use of VRT, when integrated into TAU, as added value. In terms of Demand, both therapists and patients expressed a strong interest in using VRT and saw opportunities to use VRT more broadly in the respective facilities. For therapists, facilitators that contributed to Implementation included the high level of social support, for patients the experience that VRT was not complex to use. Therapists felt well supported in practical terms (training, materials, service, knowledge) which enabled them to integrate VRT into daily work as intended. Training, monthly supervision, and a trial period appear effective implementation conditions for VRT. Adaptation by therapists and patients was good: they deployed VRT to patients’ personally set goals, while scripts proved unnecessary. To enhance integration and to maximize effectiveness, completion of the CRA communication skills module (e.g., feedback rules) by patients is a prerequisite for starting VRT.

VRT was considered broadly applicable within mental health and social care. It was feasible to provide VRT in conjunction with TAU. Due to missing data in therapists’ version, it was not possible to calculate differences between measurements. However, from the interviews, a clear trend of increase in efficacy emerges. Both therapists and patients indicated that the effects of using VRT were clearly noticeable. Patients’ self-efficacy, measured with a Likert-type scale, increased while practicing with VRT.

A single patient reported motion sickness and perceiving a virtual reality mask as oppressive. The lack of a permanent treatment room where the VR-device was set up was considered a major barrier by therapists. Each cycle of setting up, checking, and taking off the VR device takes the therapist 15–30 min of unbillable time. Cost-effectiveness should be considered given the substantial costs of training as well as hard- and software licensing. To minimize costs, two clinics shared one device. This meant that VRT could not be provided at both sites concurrently, and planning and transportation was required. We communicated the technical challenges experienced to the developer of DIVR. Several MIDI items came close to the cutoffs set for facilitators (≥ 80% agreement) and thus could still positively influence the implementation process.

To tailor VRT to diverse treatment goals and environment, some therapists and patients suggested identifying the following before the start of the VRT: the patient’s specific expressions for substance use, triggers for use, use venues, substance supply routes, whom the patient conflicts with, and the words that are helpful to the patient. Since these were also mentioned as potential future enhancements, a follow-up study should investigate the activation of all senses by also adding smells, sounds, and touchable objects and expansion of choice in virtual environments and shady characters/user friends. It is also important that therapists continue to work with the VR set on a regular basis to stay practiced.

Our study had several strengths, such as combining quantitative and qualitative data, and including both the therapist perspective and the patient perspective. In addition to showing what the key facilitators are for implementing VRT in TAU, our study also unexpectedly shows how VRT can be best implemented in regular addiction treatment. Because of our approach, few barriers were mentioned by therapists and patients. However, the perspectives on organization level, like costs and facilities, were not measured and could be a substantial barrier in considering the use of VRT. This is an important factor to consider for organizations looking to deploy VRT.

Another limitation of this study, random sampling, was not employed. It is possible that therapists and patients with positive VRT expectations were overrepresented in this study. In addition, the study took place over a limited number of sessions (three on average) and did not predetermine the frequency with which VRT sessions should be offered. Moreover, the total internal consistency of the MIDI in our sample was good to excellent. However, the sub-scales vary from low to excellent and some of the items may therefore represent different aspects within the subscale. Furthermore, self-efficacy was measured by calculating averages of the extent to which patients felt competent with respect to the skill prior and after VRT practice. Having worked with differences in therapeutic goals and a limited number of practice sessions, we recommend that a follow-up study investigates self-efficacy based on a larger number of per-patient exercise sessions. We expect that this will further enhance self-efficacy. To generalize findings, research should be extended to other institutions and populations in addiction care. We recommend focusing on the cognitive behavioral therapy (CBT) framework because it seems to have the most potential when it comes to effectiveness but also feasibility.

To our knowledge, this is the first study that addressed the feasibility of VRT integrated with TAU in addiction care. Therapists and patients expressed that VRT has an additional value over TAU-only. VRT may augment TAU. Training, monthly supervision, and a trial period seem key conditions for effective VRT implementation. Future study is required to determine the (cost-)effectiveness of VRT in addiction care.

## Appendix

Table 4

**Table 4:** General areas of focus in feasibility studies: patients and therapists Cronbach’s alpha and mean (*SD*)

**General areas of focus in feasibility studies (**Bowen et al., 2009**)**	**Patients expectations**	**Patients experiences**	**Total**
**Acceptability, **α	0.378 (2 items)	0.854 (16 items)	0.824 (18 items)
**Implementation, **α	N/A	1 item	N/A
**Limited-efficacy, **α	0.906 (11 items)	1 item	820 (12 items)
**Total, **α	0.913 (13 items)	0.880 (18 items)	0.860 (31 items)
**Total, mean (**SD**)**	3.81 (0.31)	4.16 (0.09)	4.10 (0.11)
**General areas of focus in feasibility studies (**Bowen et al., 2009**)**	**Therapists expectations**	**Therapists experiences**	**Total**
**Acceptability**	0.429 (3 items)	0.797 (15 items)	0.807 (18 items)
**Implementation**	N/A	0.385 (6 items)	0.385 (6 items)
**Practicality**	N/A	0.552 (9 items)	0.552 (9 items)
**Adaptation**	N/A	0.231 (2 items)	0.231 (2 items)
**Limited-efficacy**	0.841 (6 items)	1 item	0.822 (7 items)
**Total, **α	0.847 (9 items)	0.772 (33 items)	0.864 (42 item)
**Total, mean (**SD**)**	3.75 (0.27)	4.20 (0.09)	3.81 (0.03)
